# Role of Epigenetic Modifications in Inhibitory Immune Checkpoints in Cancer Development and Progression

**DOI:** 10.3389/fimmu.2020.01469

**Published:** 2020-07-14

**Authors:** Reem Saleh, Salman M. Toor, Varun Sasidharan Nair, Eyad Elkord

**Affiliations:** ^1^Cancer Research Center, Qatar Biomedical Research Institute (QBRI), Hamad Bin Khalifa University (HBKU), Qatar Foundation (QF), Doha, Qatar; ^2^Biomedical Research Center, School of Science, Engineering and Environment, University of Salford, Manchester, United Kingdom

**Keywords:** cancer, immune checkpoints, epigenetics, DNA methylation, histone modifications, therapeutic targets

## Abstract

A balance between co-inhibitory and co-stimulatory signals in the tumor microenvironment (TME) is critical to suppress tumor development and progression, primarily via maintaining effective immunosurveillance. Aberrant expression of immune checkpoints (ICs), including programmed cell death protein 1 (PD-1), cytotoxic T-lymphocyte-associated protein 4 (CTLA-4), T cell immunoglobulin and mucin-domain containing-3 (TIM-3), lymphocyte-activation gene 3 (LAG-3) and T cell immunoreceptor with Ig and ITIM domains (TIGIT), can create an immune-subversive environment, which helps tumor cells to evade immune destruction. Recent studies showed that epigenetic modifications play critical roles in regulating the expression of ICs and their ligands in the TME. Reports showed that the promoter regions of genes encoding ICs/IC ligands can undergo inherent epigenetic alterations, such as DNA methylation and histone modifications (acetylation and methylation). These epigenetic aberrations can significantly contribute to the transcriptomic upregulation of ICs and their ligands. Epigenetic therapeutics, including DNA methyltransferase and histone deacetylase inhibitors, can be used to revert these epigenetic anomalies acquired during the progression of disease. These discoveries have established a promising therapeutic modality utilizing the combination of epigenetic and immunotherapeutic agents to restore the physiological epigenetic profile and to re-establish potent host immunosurveillance mechanisms. In this review, we highlight the roles of epigenetic modifications on the upregulation of ICs, focusing on tumor development, and progression. We discuss therapeutic approaches of epigenetic modifiers, including clinical trials in various cancer settings and their impact on current and future anti-cancer therapies.

## Introduction

Epigenetics involves heritable and long-term changes in gene expression, which are mediated by various mechanisms, without altering the DNA sequence. In physiological and pathological settings, epigenetics plays profound, and ubiquitous roles in the regulation of gene transcription (1, 2). Epigenetic alterations in genes encoding tumor suppressors, suppressive cytokines and inhibitory immune checkpoints (ICs) can lead to impaired activation of anti-tumor immunity, tumor growth, immune escape and drug resistance, and significantly contribute to cancer development and progression (3, 4). Genetic and epigenetic modifications acquired by the tumor microenvironment (TME) play an indispensable role in tumorigenesis and result in uncontrolled growth of malignant cells (5). As cancer cells divide, they acquire genetic and epigenetic alterations giving rise to new cancer clones with different genetic and epigenetic make-ups, and inheritable traits favoring growth and survival of malignant cells (4).

The contribution of ICs to cancer pathogenesis and progression is well-recognized and has rationalized the development of monoclonal antibodies that target ICs and their ligands for cancer therapy (6, 7). Inhibitory ICs and their ligands are immunomodulatory molecules, and their physiological expression is crucial to maintain immune hemostasis and immunosurveillance to avoid potential risks of autoimmunity (8). Over expression of inhibitory ICs has been recognized as one of the major contributing factors to cancer development and progression, as well as autoimmune/chronic inflammatory diseases. Inhibitory ICs, including cytotoxic T-lymphocyte-associated protein 4 (CTLA-4), programmed cell death protein 1 (PD-1), T cell immunoglobulin and mucin-domain containing-3 (TIM-3), lymphocyte-activation gene 3 (LAG-3) and T cell immunoreceptor with Ig and ITIM domains (TIGIT), can negatively influence the activation of antigen-presenting cells (APCs) and T effector cells (Teffs), and enhance the function of T regulatory cells (Tregs) and myeloid-derived suppressive cells (MDSCs) (9).

Immune checkpoints and their ligands are differentially expressed by immune cells (Table 1). The binding of CTLA-4 to CD80 and CD86 causes inhibitory signals toward T cell activation (10). PD-1 is expressed by multiple types of immune cells, including activated T cells. Upon its interaction with its ligands, programmed cell death-ligand 1 or 2 (PD-L1 or PD-L2), inhibitory signals are generated to inhibit T cell activation/proliferation (11, 22, 23). The interaction between TIM-3 and galectin-9 has also been reported to suppress T cell function (14). The binding of LAG-3 to its ligand reduces antigen-specific CD4^+^ Teff responses and suppresses cytokine production (24–26). The interaction between TIGIT and its ligands inhibits Teff activation (19–21).

**Table 1:** Expression of immune checkpoints and their ligands.

**Immune checkpoint**	**Cellular expression**	**Ligand**	**Cellular expression**	**References**
CTLA-4	Tregs and Teffs	CD80 and CD86	APCs	(10)
PD-1	Tregs, Teffs, B cells, NK cells, mast cells, and some subsets	PD-L1 PD-L2	Tumor cells, non-lymphoid and non-hematopoietic cells	(11–13)
TIM-3	Tregs, Teffs, NK cells, and some subsets of myeloid cells	Galectin-9 CEACAM1 Soluble HMGB1 PtdSer	Some myeloid subsets; Tregs, Teffs, NK cells, and some subsets of myeloid cells; Released by tumor cells or activated DCs: Apoptotic cells	(14–16)
LAG-3	Tregs, Teffs, B cells, NK cells and DCs	MHC II	APCs	(17, 18)
TIGIT	Tregs, Teffs and NK cells	CD112 and CD155	DCs	(19–21)

Despite the success of ICIs in treating various cancer types, a large proportion of patients show low response rates due to primary or acquired resistance mechanisms. Primary resistance mechanisms are mainly dependent on the existing immune response, while acquired resistance mechanisms are governed by tumor heterogeneity/plasticity, immunosuppressive cells (including Tregs and MDSCs), T cell exhaustion and increased expression of inhibitory ICs (6, 8, 9, 27).

The overexpression ICs and their ligands, within the TME, can be mediated by different forms of epigenetic alterations, including DNA methylation, histone modifications and microRNAs (3, 28, 29). Epigenetic modifiers, including DNA methyltransferase and histone deacetylase inhibitors, can be used to revert the changes acquired during cancer onset or progression (30). The use of combined therapies targeting epigenetic modifications and ICs could serve as a highly promising therapeutic strategy to restore the physiological epigenetic profile and to boost anti-tumor immunity. In this review, we focus on the role of epigenetic modifications regulating IC expression, and promoting cancer development and progression. We discuss the different therapeutic approaches of utilizing epigenetic modifiers, including clinical trials in various cancer settings and their impact on anti-cancer therapies.

## Role of Epigenetics in Cancer Development and Progression

Epigenetics controls the transcriptional and post-transcriptional regulations of a vast array of genes, which mediate various cellular processes and functions ranging from proliferation, differentiation, invasion, survival, growth, metabolism, and immune responses (31). The development and progression of many pathological conditions, including cancer, trauma, and infectious and autoimmune diseases can be driven by aberrant epigenetic modifications (1–3, 5). Cancer was initially considered as a “genetic disease” due to gene mutations associated with loss of gene function or gene overexpression; these mutations were initially thought to be the main driving force behind disease pathogenies and progression (32). However, there is emerging evidence implicating a crucial role for epigenetics in carcinogenesis. During tumorigenesis, the epigenome is subjected to various alterations such as global changes in histone modifications, dysregulation in the non-coding RNA networks, global loss of DNA methylation, and regional hypermethylation particularly in the promoter regions of tumor suppressor genes (33). Using whole-genome sequencing, Mack et al. showed very low mutation rates, and no recurrent somatic single nucleotide variants were associated with 47 cases of pediatric brain cancer (hindbrain ependymomas) (34). In addition, the authors showed that poor prognosis of hindbrain ependymomas exhibit a CpG island methylation phenotype, which is known to induce transcriptional silencing of differentiation genes through trimethylation of lysine 27 on histone H3 (H3K27) (34). Moreover, genetic alterations in genes encoding enzymes that regulate DNA methylation and histone modifications are also responsible for predisposing individuals to cancer (35, 36). For instance, mutations in DNA methylation enzyme DNMT3a are found in ~22% of patients with acute myeloid leukemia (AML) and T cell lymphoma have been associated with poor disease outcomes (36–38). Another study showed that mutations in ten-eleven translocation 2 (TET2) methylcytosine dioxygenase, which mediates DNA demethylation, are present in ~15% of myeloid cancers (39). Mutations in genes encoding proteins that facilitate histone demethylation on H3K27, such as ubiquitously transcribed tetratricopeptide repeat, X chromosome (UTX) and additional sex combs like 1 (ASXL1), have been detected in 11% of patients with myelodysplastic syndromes and 43% of chronic myelomonocytic leukemia (40, 41). Furthermore, mutations in histone lysine acetyltransferases (HATs) and histone deacetylases (HDACs) have been associated with hematological malignancies and solid tumors (42).

Global DNA methylation and histone modifications are closely linked with cancer development and progression. The level of DNA methylation (hypo or hyper) and levels of histone methylation or acetylation can vary across different cancer types. For instance, development of breast cancer has been associated with global DNA hypermethylation, while prostate cancer pathology has been linked with DNA hypomethylation and increased active histone methylation (43). Therefore, these findings suggest that DNA methylation could occur in a tissue-specific manner depending on the TME. Certain patterns of DNA methylation, hypermethylation or hypomethylation, targeting specific genes in a particular TME could have a profound impact on cancer development and/or progression (44, 45). Based on this and since DNA hypermethylation is associated with transcriptional silencing (46), it could be anticipated that development of particular cancer types is driven by DNA hypermethylation causing reduced expression of genes related to tumor suppression and activation of anti-tumor immunity (47). On the other hand, transcriptional activation mediated by DNA hypomethylation could result in the overexpression of genes favoring tumor growth, angiogenesis, metastasis and immunosuppression, leading to the development of cancer types, which have been linked with DNA hypomethylation (44, 48).

## Role of Immune Checkpoints in Cancer Development and Progression

Increased expression of CTLA-4 and PD-1/PD-L1 and their negative correlations with overall survival (OS) in various cancer cases have been well-established (49–51). Toor et al. reported a positive correlation between tumor-node-metastasis (TNM) staging and increased expression of CTLA-4 in circulating CD4^+^ T cells of colorectal cancer (CRC) patients (52). Elevated co-expression of LAG-3 and PD-L1 in tumor tissues from triple negative breast cancer (TNBC) patients treated with adjuvant therapy has been associated with poor disease prognosis (53). Zhang et al. demonstrated that TIM-3 expression, in colorectal tumor tissues, was positively correlated with TNM staging, lymph metastasis and shorter OS (54). Furthermore, overexpression of TIM-3 on tumor-infiltrating cells showed a positive correlation with poor prognosis and shorter OS in hepatocellular carcinoma (HCC) patients (55, 56).

More recently, soluble forms of ICs/IC ligands, generated by alternative splicing and circulating in the plasma of cancer patients, have been implicated in cancer development and poor prognosis, and were suggested to serve as prognostic biomarkers. Simon et al. reported that serum soluble CTLA-4 (sCTLA-4) in pediatric patients with acute lymphoblastic leukemia can be used as a prognostic biomarker (57). High levels of sPD-1 in the plasma of patients with hepatitis B virus (HBV) were associated with high viral load and increased risk of hepatocellular carcinoma (HCC) (58). Increased levels of sPD-L1 have also been associated with poor clinical outcomes in various cancers including HCC, diffuse large B-cell lymphoma, renal cell carcinoma, and gastric and lung cancer (59–63). Additionally, poor prognosis and short OS have been linked with elevated levels of sTIM-3 in HCC patients (64). The mechanisms by which ICs and IC ligands (membrane-bound or soluble forms) mediate immunosuppression and promote tumorigenesis have been reviewed elsewhere (7, 9, 65). Collectively, the interactions between ICs and their ligands impair APC function, reduce T cell proliferation and cytokine release, induce T cell apoptosis, and enhance suppressive activity of Tregs and MDSCs (6, 27).

## Epigenetic Mechanisms Regulating the Transcription of Immune Checkpoints in Cancer

The epigenetic machinery is mainly comprised of three components: DNA methylation, histone modifications (e.g., acetylation, methylation, phosphorylation, and ubiquitylation) and non-coding RNAs/microRNAs (miRNAs) (66). In this section, we discuss how these mechanisms control the expression of IC and IC ligand genes in the TME of various cancer types.

### DNA Methylation

DNA methylation is defined as the covalent transfer of a methyl group to the C-5 position of the cytosine ring of DNA mediated by DNA methyltransferases (DNMTs) (67). DNA methylation patterns are governed by the action of DNMTs: DNMT1, DNMT3a, and DNMT3b (67, 68). Mechanistically, transcriptional silencing is mediated by a methylated cytosine by eliminating components of transcriptional regulation from their target sites (67).

**Figure 1:**
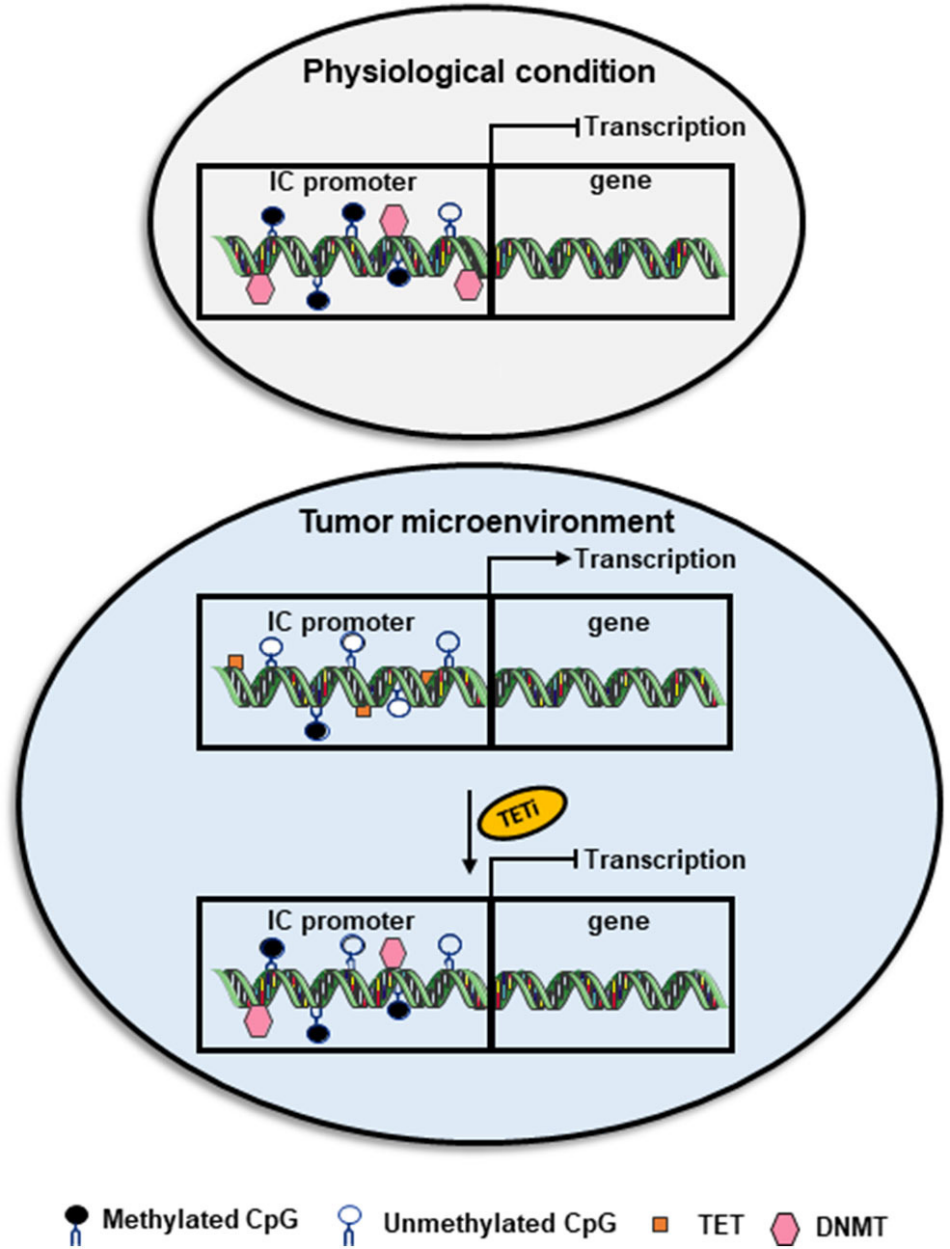
Role of DNA methylation in the transcriptional regulation of immune checkpoint expression. Under physiological conditions, the CpG islands in the promoter region of immune checkpoints are methylated by DNMTs, which leads to the transcriptional repression of ICs. However, in the TME, the activity of DNMTs could be override by the action of TETs causing TET-mediated active demethylation and favoring IC transcription. TET inhibitors could be used as a therapeutic agent to block TET-mediated active demethylation and retain the physiological condition by downregulating the transcription of genes, including ICs.

DNA methylation can be passively lost or actively driven by TET family of dioxygenases, which catalyze the oxidation of methylcytosine to hydroxymethylcytosine (69, 70). While transcriptional silencing is driven by the action of DNMT(s) leading to DNA methylation, transcriptional activation is caused by hypomethylation or demethylation facilitated by the action of TET enzyme(s) (46). Indeed, the imbalance between the activity of DNMTs and TETs can affect the expression of many genes favoring transcriptional silencing or activation during many pathological conditions, including cancer (71). For example, the upregulation of TET enzymes and downregulation of DNMTs in the circulation and tumor tissues of breast cancer (BC) and colorectal cancer (CRC) patients could be associated with DNA hypomethylation causing the upregulation of ICs/IC ligands (Figure 1) (28, 29, 72).

Restoring normal patterns of DNA methylation, especially in genes related to immune modulation and tumorigenesis, has been recognized as one of the goals for cancer therapy improvement (as described in Section Epigenetic Modifiers Targeting DNA Methylation). Aberrant DNA methylation patterns have been associated with immune evasion in cancer patients. For instance, Jung et al. reported that genomic methylation in lung and melanoma patients correlates with the immune escape signatures, independently of mutation burden and aneuploidy (73). Additionally, authors found significant negative correlations between genomic demethylation, and immunomodulatory-related pathways/immune cell markers (73), suggesting that demethylation could be responsible for silencing the transcription of these genes in patients with lung cancer and melanoma. Interestingly, they reported that global hypomethylation in these cancer patients correlated with poor clinical responses following immunotherapy, indicating that alterations in DNA methylation can be used to predict clinical benefits of immunotherapies (73). In another study, global DNA hypomethylation in human melanoma cell lines was associated with elevated expression of PD-L1, implicating a therapeutic potential for targeting PD-L1 using DNA methylation modifying agents (74).

The role of DNA methylation in regulating the expression of several ICs/IC ligands in the circulation and tumor tissues of BC and CRC patients has been addressed previously. Elashi et al. reported that increased expression of TIM-3, PD-L1, and TIGIT in the peripheral blood of both BC and CRC patients (72). DNA methylation has no role in regulating the expression of TIM-3 in the circulation of BC and CRC patients, while PD-L1 upregulation was found to be mediated by DNA hypomethylation (72). Elevated level of TIGIT in the circulation of CRC patients was mediated by DNA hypomethylation; however, DNA methylation has no role in regulating the expression of TIGIT in the circulation of BC patients (72).

Elevated expression of PD-1, CTLA-4, and TIM-3 genes in breast tumor tissues was found to be mediated by DNA hypomethylation in the CpG islands of their promoter regions (28). In the same study, authors found that the promoter regions of LAG-3 genes were completely hypomethylated in breast tumor tissues, and paired-normal tissue, suggesting that DNA methylation has no role in the upregulation of these genes in BC (28). In another study, it was reported that elevated expression of CTLA-4 and TIGIT genes in human CRC tumor tissues is driven by DNA hypomethylation (29). Additionally, authors demonstrated that DNA methylation plays no role in the overexpression of PD-1, PD-L1, galectin-9, and TIM-3 in colorectal tumor tissues (29).

A study by Marwitz et al. demonstrated that elevated expression of PD-1 and CTLA-4 in tumor tissues of non-small-cell lung cancer (NSCLC) patients is driven by DNA hypomethylation (75). However, increased expression of PD-L1 in NSCLC tumor tissues was not associated with DNA methylation (75). In contrast, elevated level of PD-L1 expression in tumor tissues of head and neck squamous-cell carcinoma (HNSCC) was a resultant of DNA hypomethylation (76). Goltz et al. demonstrated that PD-L1 promoter methylation predicts the survival rate and disease prognosis of various cancer settings, including CRC, HNSCC and AML (77–79). Another study by Rover et al. showed that increased expression of CTLA-4, PD-1, PD-L1, and PD-L2 was associated with DNA hypomethylation in patients with lower-grade gliomas (80). Altogether, these data suggest that DNA hypomethylation is responsible for increasing the expression of ICs/IC ligands in cancers; however, the set of genes regulated by DNA methylation differ from one cancer type to another.

### Histone Modifications

#### Histone Methylation

Histone methylation is another mechanism by which epigenetic modifications occur to cause transcriptional and post-transcriptional alterations in many genes, including those related to cancer development and immune evasion. These alterations affect chromatin compaction/structure, recruitment and binding of transcription factors, initiation and elongation factors with target DNAs, and RNA processing (81). Histone methylation is a dynamic process which takes place on the side-chain nitrogen atoms of lysine (K) residues, mainly on H3 followed by H4 (82). It is controlled by the activity of six major family classes of histone lysine methyltransferase complexes (KMT1, KMT2, KMT3, KMT4, KMT5, and KMT6). Lysine residues can be mono- di-, or tri-methylated by the action of KMTs (83). Lysine methylation can be reversed by lysine demethylases (KDMs), which also comprised of at least six families with distinct and overlapping functions (KDM1, KDM2, KDM3, KDM4, KDM5, and KDM6) (84, 85). The regulation of histone methylation and demethylation is a complex process (86); each KMT or KDM family consists of several enzymes that target a specific lysine residue. Additionally, different methylation states on lysine residues are controlled by different family classes of KMT or KDM, and have a different impact on transcriptional regulation.

**Figure 2:**
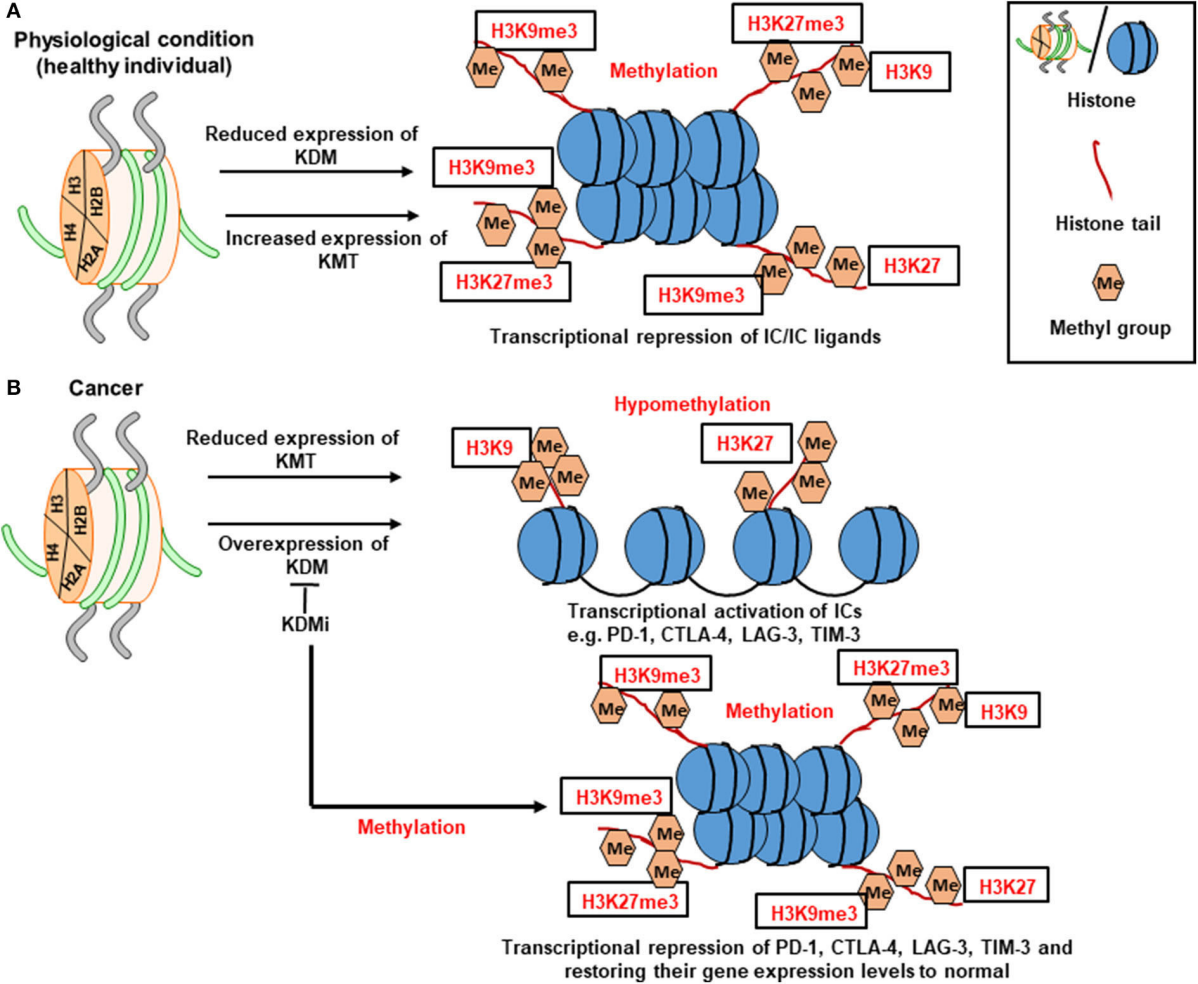
Role of histone methylation in the transcriptional regulation of immune checkpoints. Schematic diagrams simplify the complexity of gene transcription via histone methylation. Histone methylation depends on the interplay between KMTs (lysine methyltransferases) and KDMs (lysine demethylases). KMTs transfer methyl group to the histone tails. Under physiological conditions, histone methylation on the 9th and 27th lysine residues of H3 tail (H3K9me3 and H3K27me3, respectively) leads to transcriptional repression of ICs **(A)**. In tumor conditions, the low abundance of H3K9me3 and H3K27me3 leads to transcriptional activation of ICs such as PD-1, CTLA-4, LAG-3, and TIM-3. Meanwhile, utilization of KDM inhibitor (KDMi) could be beneficial in restoring the normal levels of ICs **(B)**.

Histone methylation on lysine residues appears to be a more stable mark; its loss on histones H3 and H4 causes transcriptional repression or silencing. Mono-, di- or trimethylation of lysine 4 in histone H3 (H3K4me1/2/3) and H3K36me3/me2 correlates with transcriptional activation (87, 88). On the other hand, trimethylation of lysine 9 and 27 in histone H3 (H3K9me3 and H3K27me3) correlates with repression (Figure 2A) (87, 88). The contribution of histone methylation to the regulation of IC transcription in breast and colorectal tumor tissues has been previously demonstrated. We have shown that upregulation of PD-1, CTLA-4 and LAG-3 in breast tumor tissues is associated with low enrichment of repressive histones, H3K9me3 and H3K27me3, in their promoter regions (Figure 2B) (28). In contrast, the expression of TIM-3 gene in breast tumor tissues was associated with low enrichment of H3K27me3 in its promoter region (28). In another study, increased expression of PD-1 and TIGIT in colorectal tumor tissues was shown to be associated with the low abundance of H3K9me3 in their promoter regions (29). Moreover, transcriptional upregulation of CTLA-4 in colorectal tumor tissues was found to be driven by the low abundance of H3K27me3 in its promoter region, while the low abundance of both H3K9me3 and H3K27me3 repressive histones was associated with the upregulation of TIM-3 in colorectal tumor tissues (29). Based on the above findings, it could be anticipated that targeting the activity of enzymes (KDMs) on repressive histones, H3K9me3 and H3K27me3, to maintain their trimethylation can result in the transcriptional repression of IC/IC ligand, thereby offering a therapeutic strategy for cancer treatment. The contribution of some of lysine demethylases (such as KDM3B, KDM4A, and KDM5B) to the development and/or progression of different cancer types, including breast cancer, prostate cancer and AML, have been reported, thus rationalizing the development of drugs targeting the activities of these enzymes [as reviewed in (89)].

#### Histone Acetylation

The importance of histone acetylation in regulating gene transcription and cellular processes, such as immune response, apoptosis, autophagy, cell cycle arrest, DNA damage repair, and metabolism, has been shown in cancer (86, 90). It is a highly reversible process, which involves the catalytic activity of histone acetyltransferases (HATs) and histone deacetylases (HDACs) (91). Histone acetylation occurs on lysine residues at the N-terminus induced by the activity of HATs, resulting in the removal of the basic charge at unmodified lysine residues, and leading to active transcription (92, 93). HDACs and HATs control histone acetylation act in opposite directions causing an altered structure of the chromatin, and dictate the accessibility of DNA to transcription factors (sequence-specific DNA-binding factors) and other elements of the transcriptional machinery, such as co-activators. Disrupting the equilibrium of histone acetylation or deacetylation is also reported to be associated with tumorigenesis and poor prognosis (94).

**Figure 3:**
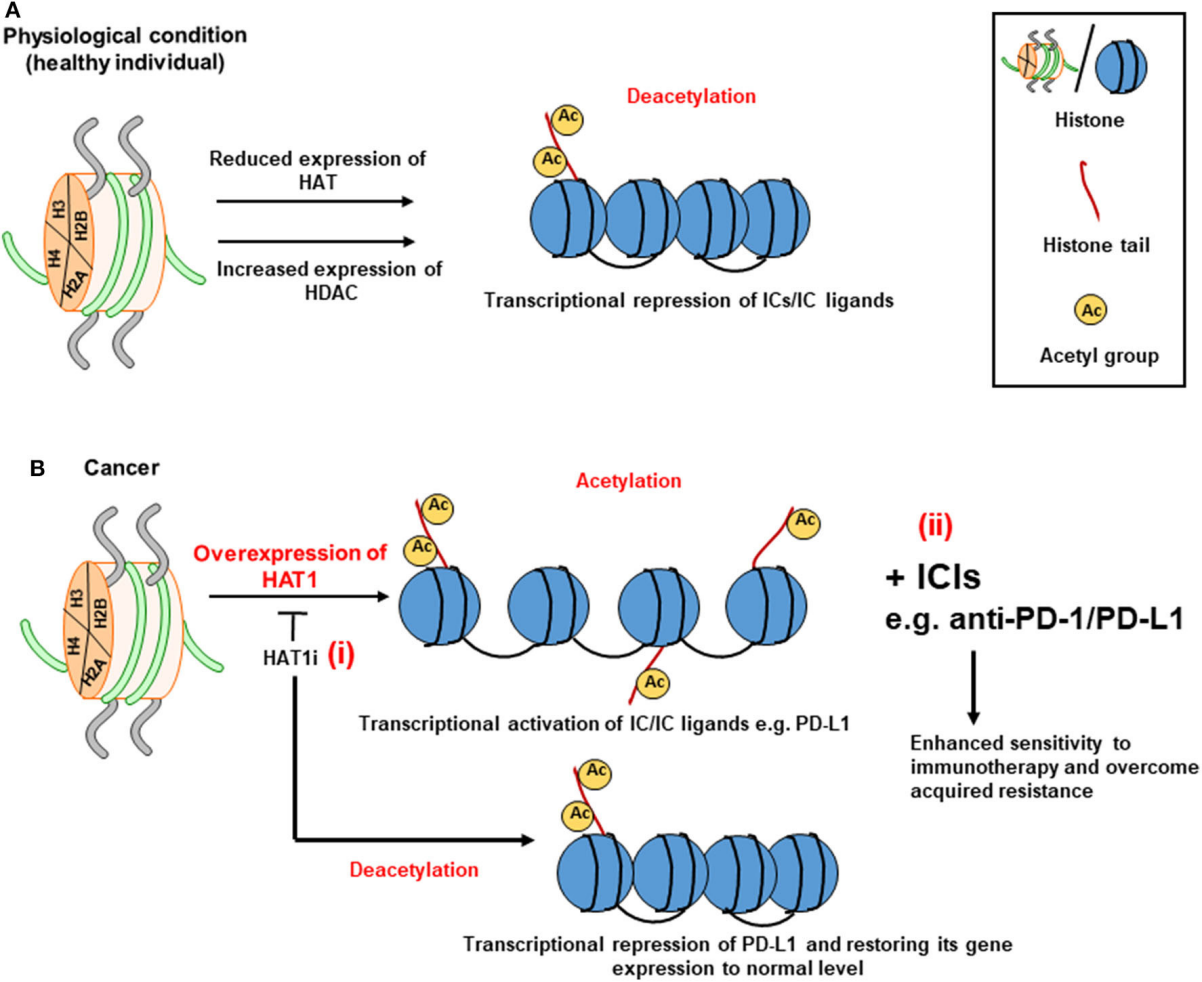
Role of histone acetylation in the transcriptional regulation of immune checkpoints. The transcriptional regulation of ICs by means of acetylation relies on the balance between HATs and HDACs on lysine residues at histone tails. A set of HDACs can keep the heterochromatin structure and downregulate the transcription of ICs in physiological conditions **(A)**. However, via tumor-acquired mechanisms, HAT activity is dominated resulting in the conversion of heterochromatin (closed chromatin) to euchromatin (open chromatin) by transferring acetyl molecules to the histone tails, thereby favoring gene transcription. Overexpression of HAT1 can lead to increased expression of PD-L1 in cancer tissues by enhancing histone acetylation. The use of HAT1 inhibitor (HAT1i) could be useful in restoring the normal expression of PD-L1- (i) **(B)**. Immune checkpoint inhibitors (ICIs) targeting PD-1/PD-L1 axis could be used in patients with aberrant expression of HAT1 and PD-L1 (ii).

HDACs stabilize the nucleosomal DNA-histone interaction causing transcriptional silencing (Figure 3A), while the action of HATs mediates transcriptional activation (Figure 3B) (91). HDACs can be divided into four classes: class I, II, III, and IV (95). The role of HDACs in cancer epigenetics and disease development is receiving an increasing attention, and targeting their activity has recently been postulated as potential therapeutic strategy for cancer treatment. HDACs repress the transcription of genes associated with immune responses and tumor suppression by restricting the accessibility of transcription factors to their binding sites and inducing a closed chromatin confirmation (96). Preclinical models of melanoma and lung adenocarcinoma showed that the expression of PD-L1 and T cell chemokines can be upregulated by HDAC inhibitors to enhance the sensitivity of the immune response to anti-PD-1/PD-L1 therapy and improve clinical outcomes (97, 98). Recently, Fan et al. reported that upregulated levels of HAT1 is associated with poor prognosis of pancreatic cancer (99). Using *in vitro* and *in vivo* models, authors also demonstrated that knockdown of HAT1 reduced the proliferation of pancreatic tumor cells, and downregulated PD-L1 expression (99). Furthermore, it was shown that PD-L1 expression positively correlated with HAT1 expression in pancreatic tumor tissues (99). Altogether, these findings suggested that HAT1 transcriptionally regulate PD-L1 expression in cancer settings, and implicated that targeting HAT1 activity could be used as a therapeutic approach for cancer treatment (99) (Figure 3B, i). Alternatively, the use of ICIs targeting PD-1/PD-L1 axis in patients with acquired resistance (97, 100) due to aberrant expressions of HAT1 and PD-L1 (99) could be beneficial in maximizing the anti-tumor immune response, enhancing the sensitivity to ICI, and overcoming resistance (Figure 3B, ii). Collectively, these findings suggest that HDACs act opposite to HATs in terms of IC regulation, and that HDAC inhibition in combination with ICIs could be beneficial in enhancing the therapeutic efficacy of cancer treatment by increasing the sensitivity of the host immune response to ICIs. This particular therapeutic strategy could be favorable for cancer patients who developed acquired resistance to ICIs.

Given the complexity of epigenetic regulations and knowing the fact that HATs and HDACs can alter the transcription of multiple target genes, it is crucial to take this into consideration during the development of HAT and HDAC inhibitors and the design of therapeutic protocols. For instance, HDAC inhibitors, valproic acid (VPA; class I HDAC inhibitor) and trichostatin-A (TSA; class I and II inhibitor), could induce apoptosis and alter the acetylation status of p53, on ETS Related Gene (ERG)^+^ prostate cancer cells (101, 102). In addition, VPA and TSA were able to repress the transcription of ERG, which its overexpression has been associated with poor prognosis and unfavorable clinical outcomes in prostate cancer patients (101).

### Long Non-coding RNAs and MicroRNAs

Long non-coding RNAs (lncRNAs) are a series of non-coding RNAs comprised of more than 200 nucleotides. lncRNAs are pointed to as potential candidates to evaluate the prognosis, diagnosis, and development of cancers, even though their capacity of protein-coding is very little (103). MicroRNAs (miRNAs) are small non-coding RNAs (19–25 nucleotides long), which complementary pair with the 3′ untranslated region of target mRNAs, resulting in the repression of transcription and/or the degradation of target mRNAs (104). Studies demonstrated that miRNAs can regulate more than 30% of human genes involved in many cellular processes, including cell cycle arrest and cell growth/proliferation/differentiation/apoptosis (105–107).

miRNAs in various cancers can influence the transcriptional regulation of immunomodulatory genes, including ICs and their ligands (108). Wei et al. showed that transfection of human CD4^+^ T cells with miR-138 abolished the expression of CTLA-4, PD-1 and FoxP3 expression in glioma mouse models (109). Another miRNA with tumor-suppressive functions is miR-28. Li et al. reported that the expression of miR-28 is downregulated ~30% in exhausted PD-1^+^ T cells from melanoma patients (110). Authors reported that miR-28 inhibits the expression of the TIM-3 and PD-1 in T cells upon the binding to their respective 3′ UTRs (110). In ovarian carcinoma, signaling pathways mediated by the interactions of CTLA-4 with CD80 and PD-1 with PD-L1 are negatively regulated by mi-R424(322) (111), suggesting the importance of mi-R424 in the downregulating of CTLA-4 and PD-1 signaling pathways. In support of this, it was shown that high levels of mi-R424(322) in tumors are positively correlated with progression-free survival in patients with ovarian carcinoma (111). More recently, Richardsen et al. demonstrated that low levels of miR424-3p in prostate cancer (PC) tissues associated with an aggressive phenotype of PC, poor disease prognosis and low survival rate (112). Authors also reported a negative correlation between CTLA-4 expression and miR424-3p expression in PC tissues (112), highlighting the role of miR424-3p in regulating CTLA-4 expression in PC as it has been reported in other cancer types (111, 113).

A study by Cortez et al. demonstrated that PD-L1 expression in NSCLC is negatively regulated by p53 via miR-34, suggesting that miRNA delivery could serve as a novel therapeutic approach for lung cancer therapy (114). Studies indicated that miRNAs can affect the progression of AML by modulating the expression of target genes such as TIM-3. Based on bioinformatics, it was predicted that miR-330-5p may silence the transcription of TIM-3 in the AML cell line, HL-60 (115). Acquired resistance against anti-PD-1 therapy has been associated with the upregulation of TIM-3 on T cells in lung cancer and HNSCC patients (116, 117). Another study by Oweida et al. showed that response to anti-PD-L1 mAb and radiotherapy was compensated by the increased expression of TIM-3 on CD8^+^ T cells and Tregs, associated with tumor relapse, poor survival rate in a mouse model of head and neck tumor (118). Collectively, these studies suggest that the use of ICIs in combination with miRNA therapy to target alternative ICs, could be beneficial in preventing the development of acquired resistance in response to anti-PD-1 or anti-PD-L1 therapies.

In lymphoma, the expression of CTLA-4, PD-1, PD-L1, TIM-3 and LAG-3 are negatively regulated by miR-146 (119). The expression of PD-L1 on tumor cells and the suppression of anti-tumor immunity in human lung cancer are negatively regulated by miR-200 (120). Another miRNA with tumor suppressive functions is miR-34a. Its expression is induced by p53, which in turn suppresses the expression of PD-L1 (120). In line with this, it was reported that low levels of miR-34a in AML and NSCLC are positively correlated with the overexpression of membrane–bound PD-L1 (53, 114). Overexpression of PD-L1, and low levels of p53 and miR-34a have been associated with poor clinical outcomes in patients with NSCLC (120). On the other hand, overexpression of miR-34a can dysregulate the activation of PD-1/PD-L1 signaling pathway, causing the reversal of CD8^+^ T cell exhaustion, and triggering T cell activation and cytokine expression, such as IFN-γ and TNF-α (121). In CRC, low levels of miR138-5p, a tumor suppressive miRNA, positively correlates with advanced disease stages, lymph node metastasis and poor clinical outcomes (122). miR138-5p negatively regulates PD-L1 expression in CRC, which is associated with reduced cell proliferation and cell cycle progression (122).

**Figure 4:**
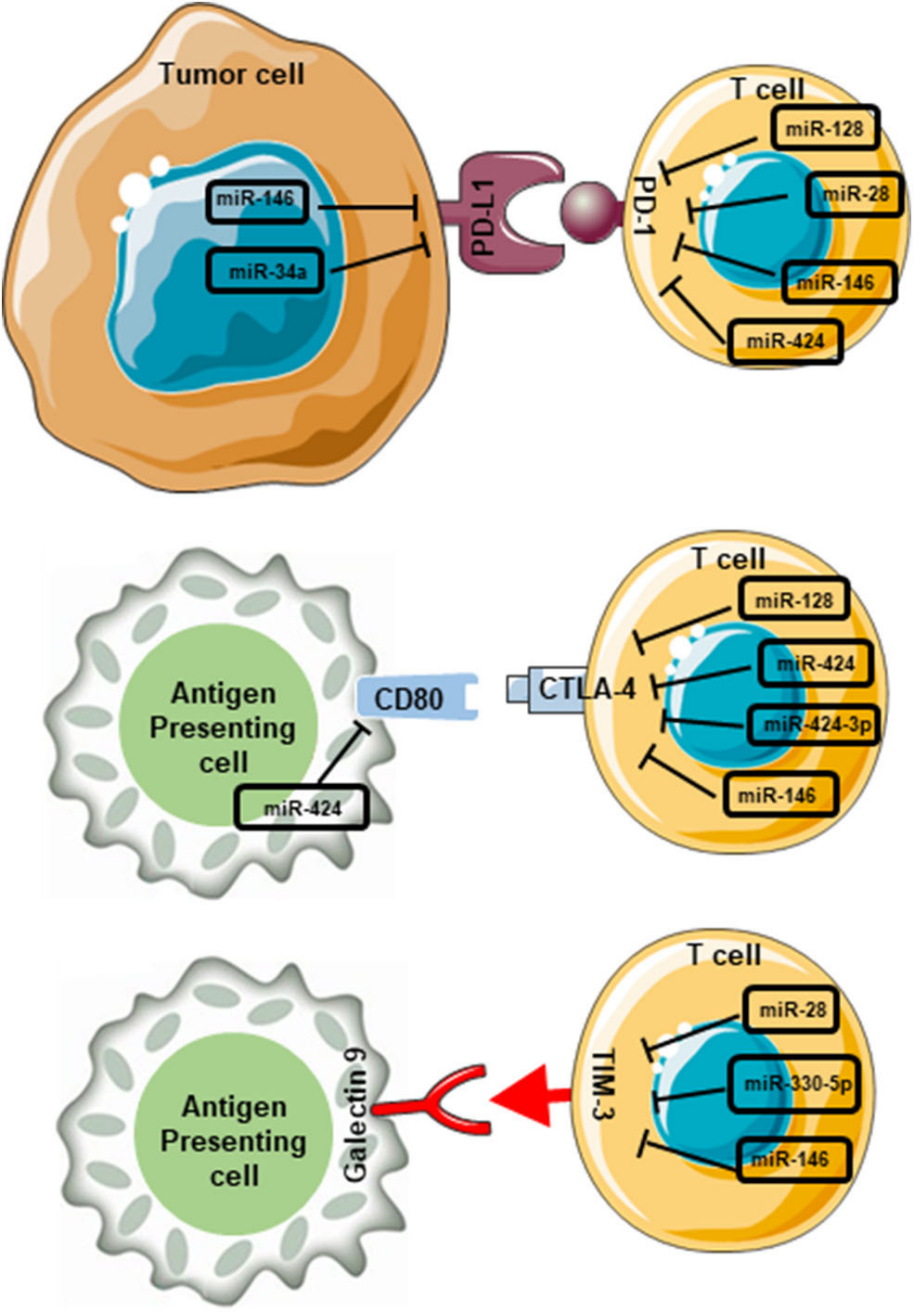
miRNA-mediated interruption of interactions between immune checkpoints and their ligands in the tumor microenvironment. miRNAs which contribute to the blockade of PD-1/PD-L1 interactions are miR-146, miR-34a, miR-128, miR-28, miR-146, and miR-424. miR-146 and miR-34a expressed on tumor cells, and miR-128, miR-28, miR-146, and miR-424 expressed on T cells. Likewise, miR-424 expressed on APCs, and miR-128, miR-424, miR-424-3p, and miR-146 expressed on T cells interfere with CD80/CTLA-4 interactions. Furthermore, miR-28, miR-330-5p, and miR-146 expressed on T cells interfere with TIM-3/galectin 9 interaction. These miRNA-mediated interruptions could lead to the blockade of downstream pathways, which ultimately favor anti-tumor immunity.

Collectively, these findings clearly imply the importance of miRNAs in regulating the expression of genes related to tumorigenesis, immune evasion and cancer progression. One miRNA may have several mRNA targets, and therefore could influence the function of many genes, pathways and cellular processes. The overall role of various miRNAs on the regulation of ICs and their ligands are summarized in Figure 4. The above findings also suggest the potential therapeutic benefit of including miRNAs in cancer therapy as it will be discussed below.

## Potential Therapeutic Applications of Epigenetic Modifiers for Cancer Treatment

**Figure 5:**
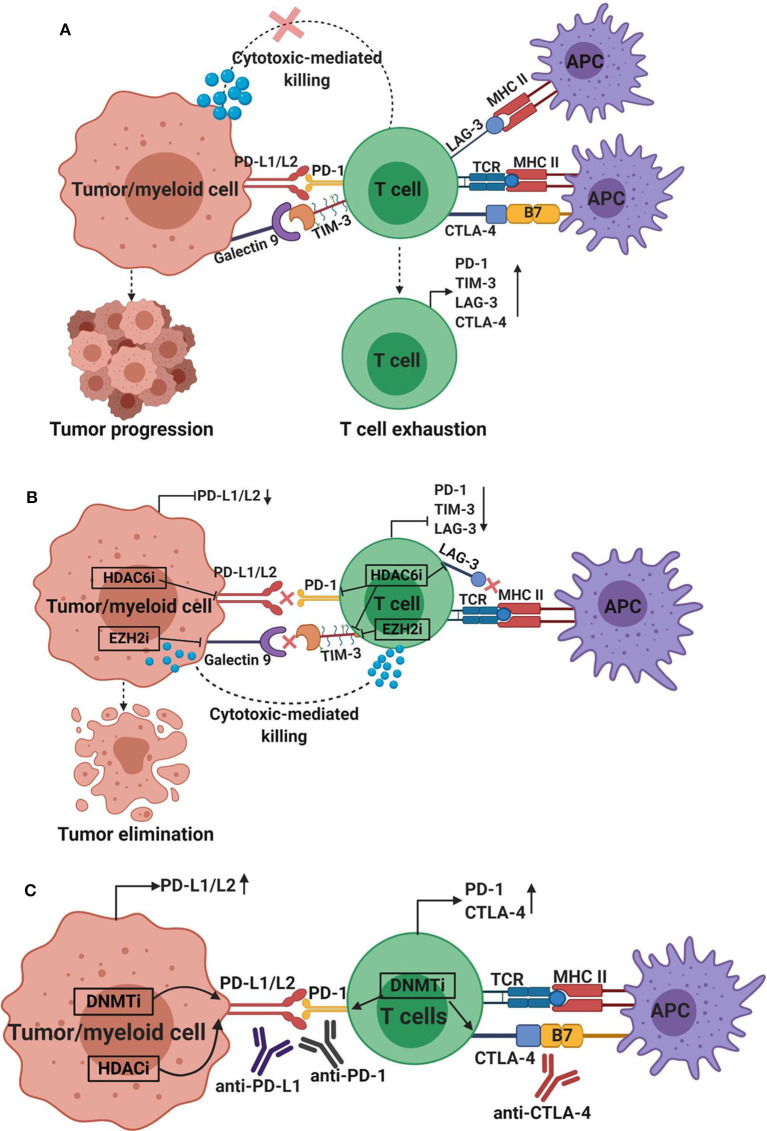
Effect of epigenetic modifiers on the expression of immune checkpoints and their ligands in the tumor microenvironment. The interaction between co-inhibitory immune checkpoints on immune cells and their ligands on tumor cells or myeloid cells results in tumor progression, immunosuppression and T cell exhaustion characterized by increased expression of immune checkpoints, including PD-1, CTLA-4, TIM-3, and LAG-3, and loss of effector functions, such as cytokine release and cell-mediated cytotoxicity. The interactions between PD-1, TIM-3, CTLA-4, and LAG-3 on T cells with their respective ligands PD-L1/PD-L2, galectin-9, B7 ligands or MHC II on tumor cells/myeloid cells or APC, generate signals that inhibit T cell activation/proliferation **(A)**. Depending on the tumor microenvironment and tumor type, the application of epigenetic modifiers can downregulate or upregulate the expression of immune checkpoints and their ligands. The application of HDAC6 inhibitor (HDAC6i) can downregulate the expression of PD-L1/2, PD-1, TIM-3, and LAG-3, and EZH2 inhibitor (EZH2i) can downregulate the expression of galectin-9 and TIM-3 **(B)**, indicating the potential benefits of using these modifiers to enhance anti-tumor immune responses and promote tumor cell killing. On the other hand, application of DNMT inhibitor (DNMTi; azacytidine or decitabine) can upregulate the expression of PD-L1/2, PD-1 and CTLA-4, and HDAC inhibitor (HDACi; vorinostat; or panobinostat) can upregulate the expression of PD-L1/2 **(C)**, suggesting the potential benefit of combining epigenetic modifies with immune checkpoint inhibitors, such as anti-CTLA-4, anti-PD-1 or anti-PD-L1, to increase the sensitivity of the host immune response and promote more potent anti-tumor immunity.

Studies have shown that cancers exploit epigenetic mechanisms mainly in two ways: (1) to delineate the normal transcriptional regulation of gene expression to assist tumor progression; and (2) to deactivate anti-tumor immune responses, and regulate oncogenes and tumor suppressor genes. Dysregulated transcription of co-activators or suppressors of oncogenes/proto-oncogenes and tumor suppressor genes leads to the development of various human cancers. Hypomethylation leads to genomic instability, while hypermethylation may lead to silencing of tumor suppressor genes (123). Therefore, the development and use of epigenetic modifiers aiming to modulate the activity of enzymes involved in these epigenetic pathways, including DNMTs, TETs, HATs and HDACs, may offer therapeutic benefits (96, 124). However, it is important to consider the complexity of epigenetic regulations and take into consideration the tumor type, nature of the TME, and all the target genes that can be altered upon the inhibition of epigenetic mediators (DNA/histone modifiers) during the development of epigenetic drugs and the design of therapeutic protocols. The communication between immune cells and tumor cells via IC/IC ligand interactions results into immunosuppression and tumor progression (Figure 5A). Some epigenetic drugs can be used to enhance anti-tumor immunity by downregulating the expression of ICs/IC ligands (99) (Figure 5B), while others could be used in combination with ICIs to improve the sensitivity of the host response to therapy by upregulating the expression of IC ligands (97, 100) (Figure 5C). This should be useful during the assignment of therapeutic protocols for cancer patients.

### Epigenetic Modifiers Targeting DNA Methylation

DNA methylation may lead to silencing of suppressor genes, such as TP53 and CDKN2A, thereby increasing susceptibility to cancer onset. Inhibition of DNMTs has been shown to correlate with increased expression of tumor suppressor genes and reduction in tumorigenicity (125). Hypomethylating agents, which inhibit DNMT, target the methylation patterns of tumor cells to reinstate normal methylation signatures. DNMT inhibitors (DNMTis), such as 5-azacytidine (AZA/5AC) and decitabine, have been developed to inhibit and degrade DNMTs, reverse hypermethylation and promote transcriptional activation (126). Several signaling pathways such as those related to double-stranded RNA (ds-RNA) response, type I interferon response and apoptosis are induced upon the application of DNMTis. In a preclinical melanoma model, DNMTi treatment was able to increase the sensitivity to anti-CTLA-4 therapy by affecting hypermethylated endogenous retrovirus genes (127). DNMTis, azacytidine and decitabine, have also been shown effective in increasing PD-L1 and PD-L2 levels in melanoma (97) (Figure 5C). Animal studies showed that combining azacytidine or decitabine with anti-CTLA-4 in ovarian cancer and melanoma is beneficial in improving the immune response to anti-CTLA-4 and reducing tumor burden (128, 129). Altogether, these data rationalized the use of DNMTis in combination with ICIs to maximize the therapeutic efficacy and clinical outcomes in cancer patients (129). Azacytidine and decitabine serve as the most commonly used DNMTi in oncology for the treatment of chronic myelomonocytic leukemia (CMML), myelodysplastic syndromes (MDS) and AML (130). Treatment of MDS with decitabine increased the mRNA expression of PD-1, its ligands (PD-L1 and PD-L2) in addition to CTLA-4 (131), rationalizing the potential synergy between DNMTi and ICIs in enhancing the therapeutic efficacy of combined treatment, as hypomethylation may increase the expression of ICs/IC ligands, and subsequently sensitize tumor cells to the ICIs (Figure 5C). Several trials are currently underway to investigate DNMTi use in treating different solid malignancies (132–134). However, DNMTi therapies are frequently associated with severe side effects, no or partial treatment responses and therapy resistance in a significant patient cohort. Therefore, identifying novel, more specific targets against DNMTi are currently being explored.

TET-mediated DNA demethylation contributing toward developmental processes including disease progression and its dysregulation may lead to tumorigenesis (135). As previously discussed in Section DNA Methylation, TET-mediated DNA demethylation could be associated with the overexpression of IC/IC ligand in the circulation and tumor tissues of patients with breast and colorectal cancers (28, 29, 72). Therefore, the inhibition of TET activities can have a therapeutic potential and could be beneficial in restoring the normal transcriptomic expression and methylation patterns of IC/IC ligand. Furthermore, TET mutations have been associated with various hematological malignancies; however, specific TET protein inhibitors have not been tested till present in clinical oncology (124, 136). Nevertheless, upstream targets for TET-associated pathways have been identified in different malignancies and have been the focus of numerous preclinical studies. For instance, mutations in genes encoding isocitrate dehydrogenase 1 and 2 lead to TET1 inactivation in gliomas (137).

### Epigenetic Modifiers Targeting Histone Modifications

HATs modify chromatin histones to exert their effects of epigenetic modulation of gene transcription, and are dysregulated in various human diseases including cancers (138). For instance, HAT1 has been implicated in the transcriptional upregulation of PD-L1 in pancreatic cancer (99). Additionally, it has been demonstrated that knockdown of HAT1 reduced the proliferation of pancreatic tumor cells, and expression of PD-L1 (99). These findings suggest that targeting HATs could be beneficial in reducing the expression of IC ligands, and ultimately could have clinical benefits for cancer patients. However, in contrast to HDAC inhibitors (HDACis), HAT inhibitors (HATis) are yet to be explored in preclinical/clinical trials (139). Significance of HATi is mainly overshadowed by the well-established HDACi. However, studies have shown that HATi can be equally potent blockers of tumorigenesis as HDACi (140).

Vorinostat (class I and II) and romidepsin (class I) are FDA-approved HDACi commonly used to treat several malignancies. HDACi promote acetylation of histones and modulate expression of ~2–10% of cellular genes via effects on chromatin structure and transcription factor/cofactor binding, leading to either increase or decrease in expression (141). It has been shown that the use of vorinostat and panobinostat (pan-HDACi) is able to increase the expression of PD-L1 in TNBC, and PD-L1 and PD-L2 in melanoma by altering chromatin compaction on their promoter regions (100, 142) (Figure 5C).

Other non-canonical effects of HDACis on the regulation of immune responses are also evident from numerous studies. Reports have shown that HDACis can inhibit tumor growth and enhance the host immune response against cancer cells via the suppression of Tregs and FoxP3 expression (143), upregulation of NK cell activating ligands, MHC molecules (class I and II), enhancement of NK and CD8^+^ T cell cytotoxicity and production of pro-inflammatory cytokines (143–145). Class II HDACi (entinostat) in combination with DNMTi (azacytidine), anti-CTLA-4 and anti-PD-1 mAbs improved treatment outcomes, associated with tumor regression and absence of metastasis in murine models of CT26 colorectal tumors and 4T1 metastatic breast cancer (146). The number of tumor-infiltrating FoxP3^+^ Tregs was significantly reduced upon treatment with epigenetic modulators, compared to ICIs; however, the effect of epigenetic modulators on tumor-infiltrating CD8^+^ T cell number was similar to that induced by ICIs alone (146). A study by Orillion et al. demonstrated that the use of entinostat (class I HDACi) suppressed the function of MDSC and enhanced the anti-tumor effects of anti-PD-1 therapy in murine models of lung and renal cell carcinoma, suggesting a rationale for combining HDACi and ICIs in clinical trials (147). Other studies showed that using HDACis in combination with anti-PD-1 therapy enhances the anti-tumor immune response, reduces tumor burden and increases survival in murine tumor models (97, 100). Woods et al. demonstrated that the treatment with HDACi increases the expression of PD-L1 in murine melanoma mouse model, thereby enhancing the sensitivity to anti-PD-1 therapy and overcoming resistance to therapy (97). Similarly, Briere et al. showed that class I/IV HDACi increased the expression of PD-L1 in syngeneic tumor models, and demonstrated that the HDACi in combination with anti-PD-L1 enhanced the anti-tumor immune response compared to their use as a monotherapy (148).

Preclinical studies demonstrated that the upregulation of ICs/IC ligands can be epigenetically modulated. Inhibition of HDAC activity has been reported to modulate PD-L1 expression in chronic lymphocytic leukemia (CLL) and melanoma (97, 149). Recently, Knox et al. demonstrated that the use of HDAC6i significantly reduced the upregulation of PD-L1 and PD-L2 (Figure 5B) in SM1 murine melanoma model, increased expression of IFN-γ and IL-2, and improved survival rates (150). Notably, Kim et al. have recently shown that CG-745, a class I and HDAC6i, induced IL-2 and IFN-γ expression, promoted cytotoxic T cell/NK cell proliferation and inhibited Treg proliferation, which consequently promoted effects of anti-PD-1 therapy in syngeneic mouse models (151). Furthermore, Laino et al. showed that HDAC6 inhibition downregulated the expressions of TIM-3, PD-1, and LAG-3 on expanded T cells from the circulation of melanoma patients (152), indicating the potential benefits of blocking HDAC6 activity to alleviate T cell suppression. Additionally, Bae et al. showed that HDAC6 inhibition reduced the expression of PD-L1 on multiple myeloma bone marrow cells and PD-1 expression on CD8^+^ T cells (153) (Figure 5B).

The regulation of gene transcription by histone methylation/demethylation is a complex process, which is controlled by the activity of different family classes of enzyme complexes, KMTs (83) and KDMs (84, 85). Different classes of KTMs and KDMs act on different lysine residues on histone H3 or H4 and regulate the expression of various target genes. H3K27me3 is known as a transcriptional repressor for many genes including those associated with tumor resistance to therapy (147). Methylation of H3K27me3 is positively regulated by polycomb repressive complex 2 (PRC2), a member of the KMT family, and its enzymatic subunit, enhancer of zeste homolog 2 (EZH2) (147, 154, 155). Together, these data suggest that targeting EZH2 could interfere with the transcriptional repression mediated by H3K27me3, and therefore overcome tumor resistance to therapy and improve disease outcomes. EZH2 has been implicated in various cancers including melanomas, ovarian, prostrate, and breast cancers (136, 156).

Increased expression of EZH2 has been associated with the development of acquired resistance against recombinant IL-2 (rIL2) and anti-CTLA-4 therapies in melanoma mouse model (154). On the other hand, co-inhibition of EZH2 with rIL-2/anti-CTLA-4 immunotherapies resulted in the downregulation of PD-L1 expression in melanoma cells, increased number of intratumoral PD-1^low^TIM-3^low^LAG-3^low^CD8^+^ T cells expressing high levels of IFN-γ and suppression of tumor growth (154). Using *in vitro* and *in vivo* models, EZH2 activity has been reported to be responsible for the progression of hepatocellular carcinoma by enhancing the expression of galectin-9, TIM-3 ligand, via the trimethylation of H3K27 (157), suggesting that inhibition of EZH2 could be useful for targeting galectin-9 and TIM-3 expression (Figure 5B). Collectively, these results suggest the potential therapeutic benefits of targeting EZH2 in cancer to downregulate IC/IC ligand expression and enhance anti-tumor immunity, and rationalized for the development of histone methylase inhibitors targeting EZH2 in cancer (136), which are currently under different clinical trials for treating different malignancies.

### Long Non-coding RNAs and microRNAs as Potential Therapeutic Strategies for Cancer

A recent study by Ma et al. showed that lncRNA, lnMX1-215, negatively regulates PD-L1 and galectin-9 in HNCC and its overexpression significantly reduces tumor cell proliferation /metastasis *in vitro* and *in vivo* (158). Authors proposed lnMX1-215 as a potential therapeutic target for HNCC by interfering with PD-1/PD-L1 and TIM-3/galectin-9 signaling pathways and restoring anti-tumor immunity (158).

miRNAs are aberrantly expressed in many types of cancer and malignancies; they regulate the expression of tumor suppressor genes, oncogenes, ICs and immune checkpoint ligands (108). miRNAs have a great advantage over other non-coding RNAs, and mRNAs; they are more stable in biopsy specimens and body fluids, allowing their use as biomarkers (159–161). Moreover, miRNA expression profiles are tissue-specific, which is helpful in speeding up the diagnosis of specific cancer types (160, 161). By upregulating the expression of ICs and IC ligands, miRNAs can contribute to cancer development/progression and compromise the anti-tumor immune responses (108, 162). Targeting this regulatory function of miRNAs can be used to improve clinical responses and enhance the sensitivity of cancer patients' response to immune checkpoint inhibitors (ICIs).

The single blockade of IC commonly results in the upregulation of alternative ICs, suggesting the emergence of compensatory mechanisms which ultimately leads to resistance to ICIs (27, 117, 118, 163). Single miRNA can target multiple ICs/IC ligand in multiple cell types in the same tumor tissue. Hence, this will mimic the effect of the treatment with multiple ICIs and could be used as a therapeutic agent. For instance, tumor suppressive miRNA, miR-138, can be used to reduce the expression of PD-1, and CTLA-4, induce tumor cell apoptosis and impair invasion and tumor metastasis (109, 164, 165). Zhao et al. reported SHNG14/ZEB1/miR-5590-3p positive feedback loop in diffuse B cell lymphoma (DBCL) is associated with attenuated CD8^+^ T cell activation through PD-1/PD-L1 axis, suggesting that targeting SHNG14 holds the promise of enhancing anti-tumor immunity and restrain tumor progression (166). Another therapeutic strategy that could be employed in cancer treatment is targeting the function of tumor promoting miRNAs using anti-miRNAs (167, 168).

The use of miRNA as a monotherapy is not beneficial and may result in adverse immunologic effects, given that each miRNA can act on multiple target genes, including those encoding immune modulatory molecules (169). Therefore, small doses of anti-miRNAs can be used in combination with chemotherapy or immunotherapies to minimize the risk of adverse effects (108). In addition, miRNAs could be more beneficial if used in combination with ICIs. They may increase the sensitivity of the host immune response to a particular ICI and overcome tumor acquired resistance. In other words, this combination therapy would convert non-responder patients into responders. For instance, Li et al. demonstrated that miR-28 induces T cell exhaustion by upregulating the expression of PD-1, TIM-3, and BTLA (110). This potentially suggests that use of miR-28 in addition to ICIs, especially those targeting PD-1 and/or TIM-3 could result in beneficial outcomes and enhance anti-tumor immunity. Studies have shown negative correlations between miR-138-5p and PD-L1 expression (122), miR-138 and PD1/CTLA-4 expression (109), and miR-424 and PD-L1 expression (111), suggesting that targeting these miRNAs increase the expression of ICs. Thus, we could rationalize that targeting particular miRNAs could be useful in upregulating the expression of ICs, which increases the sensitivity and efficacy of ICIs.

## Clinical Trials for Combined Therapeutic Strategies of Epigenetic Modifiers and ICIs

Epigenetic modifiers have the potential to increase the sensitivity to ICIs and restore more potent anti-tumor immune responses and enhance the clinical responses in cancer patients. Several preclinical models have supported the rationale for combining epigenetic modifiers with ICIs, and implicated the need to design clinical trials to assess the efficacy of targeting DNA methylation and HDAC activity, in combination with ICIs, in different cancers (details of ongoing clinical trials are listed in Table 2). Results from completed phase II clinical trial of pembrolizumab (anti-PD-1) in combination with azacytidine in microsatellite stable (MSS) metastatic colorectal cancer patients showed that the combined therapy had mild anti-tumor effects associated with some adverse effects such as anemia, leukopenia and constipation (171).

**Table 2:** Examples of preclinical models and ongoing clinical trials for combination therapies utilizing ICIs and epigenetic modifiers.

**Model**	**Therapy**	**Outcome**	**Clinical trial**	**Therapy in cancer patients**
Mouse ovarian cancer model	Decitabine and anti-CTLA-4	Synergistic reduction in tumor growth and prolonged survival rates (128)	NCT02915523 Phase Ib/II clinical trial in patients with chemo-resistant epithelial ovarian cancer	Entinostat (class I HDACi), together with avelumab (anti-PD-L1)
			NCT0329217 Phase Ib open label clinical trial in patients with advanced ovarian cancer or triple negative breast cancer	Histone lysine methyltransferase (BET) inhibitor with atezolizumab (anti-PD-L1)
*Ex-vivo* model for tumors from metastatic renal cell carcinoma (RCC)	EZH2 and DNMT1 inhibitors and anti-PD-L1	Synergistic reduction in tumor growth and prolonged survival rates (170)	NCT02619253 Phase II/III clinical trial in patients with advanced renal or urothelial cell carcinoma	Vorinostat (class II HDACi) with pembrolizumab (anti-PD-1)
			NCT02508870 Phase I clinical trial in patients with hypomethylating agent (HMA)-naïve myelodysplastic syndromes (MDS)	Azacytidine with atezolizumab (anti-PD-L1)
			NCT02397720 Open-Label Phase II trial in patients with refractory/ relapsed AML and Newly Diagnosed Older AML	Nivolumab (BMS-936558) in Combination With 5-Azacytidine (Vidaza) or Nivolumab With Ipilimumab in combination with 5-Azacytidine
			NCT02599649 Phase II trial in patients with myelodysplastic syndromes (MDS)	Combination of Lirilumab and Nivolumab With 5-Azacytidine
			NCT02530463 Phase II trial in patients with myelodysplastic syndromes (MDS)	Combination of nivolumab and ipilimumab with 5-Azacytidine
Mouse melanoma model	Class II HDACi (panobinostat) and anti-PD-1	Slower tumor progression and prolonged survival (97)	NCT02453620 Phase I clinical trial in patients with HER2^+^ breast cancer	Entinostat with nivolumab (anti-PD-1) and ipilimumab (anti-CTLA-4)
			NCT02395627 Phase II clinical trial in patients with ER^+^ advanced hormone therapy-resistant breast cancer	Tamoxifen with vorinostat and pembrolizumab (anti-PD-1)
Mouse chronic lymphocytic leukemia (CLL) Primary B cell isolated from CCL patients and CCL cell lines	HDAC6 inhibitor (ricolinostat)	Reduction in the expression of co-inhibitory receptors on all T cell subsets, substantially CD8^+^ effector and memory cells (149)	NCT02453620 Phase I clinical trial in patients with solid tumors; breast adenocarcinoma, invasive breast carcinoma, metastatic breast carcinoma, metastatic malignant solid neoplasm	Entinostat with nivolumab (anti-PD-1) and ipilimumab (anti-CTLA-4)
			NCT02635061 Phase Ib in patients with unresectable non-small cell lung cancer (NSCLC)	Selective HDAC6 inhibitor (ACY-241) with nivolumab (anti-PD-1)
Mouse CT26 and 4T1 tumor models	Class I HDACi (Entinostat), anti-CTLA-4 and anti-PD-1	Reduced levels of MDSCs, enhanced functions of Teffs (146)	NCT02538510 Single Arm Phase I/II clinical trial in patients with recurrent unresectable and/or metastatic squamous cell head and neck cancer and recurrent unresectable and/or metastatic salivary gland malignancies	MK-3475 combined with vorinostat and pembrolizumab (anti-PD-1)
			NCT02708680 Phase II clinical trial in patients with advanced triple negative breast cancer	Atezolizumab (anti-PD-L1) with or without Entinostat
			NCT02638090 Phase I/II trial in patients with immune therapy naïve and immune therapy pretreated stage IV NSCLC	Combination of with pembrolizumab and Vorinostat
Mouse CT26 and 4T1 tumor models	Class I HDACi (Entinostat), DNMTi, anti-CTLA-4 and anti-PD-1	Reduced levels of MDSCs, enhanced functions of Teffs (146)	NCT02032810 Phase I in patients with Unresectable stage III/IV melanoma	Panobinostat and ipilimumab (anti-CTLA-4)
			NCT01928576 Phase II clinical trial in patients with metastatic non-small cell lung cancer	Azacytidine and entinostat with nivolumab (anti-PD-1)

## Conclusions and Future Directions

Epigenetic modifiers have thus seen important advances in recent years, and currently several are being explored in combination with established ICIs in various clinical trials (172). The rationale for these studies is based on the recent success of ICIs in different cancers, and the unresponsiveness of some cancer patients to current therapies, which is believed to be associated with acquired resistance mechanisms mediated by epigenetic alterations. However, it is noteworthy that several epigenetic enzymes also contribute to cancer progression via other non-epigenetic mechanisms, and ultimately combination therapies to tackle cancer on different fronts with more targeted precision medicine approaches may provide the most effective anti-cancer therapy. It is important to note the complexity of epigenetic regulations while designing epigenetic drugs, and take into consideration all the target genes, which their transcription can be regulated by a specific epigenetic drug. In addition, epigenetic modifiers may have different effects on cancer cells and different types of immune cells, depending on the target genes (173). Further investigations are required to assess the clinical efficacy of using miRNAs in combination with ICIs, and the risk of adverse effects related to toxicity and potential development of autoimmunity.

## Author Contributions

RS wrote the manuscript. ST and VS assisted in writing and reviewing the manuscript. EE conceived the topic, wrote, and revised the manuscript. All authors were involved in the final approval of the manuscript.

## Conflict of Interest

The authors declare that the research was conducted in the absence of any commercial or financial relationships that could be construed as a potential conflict of interest.

## Abbreviations

APC: Antigen-presenting cell
BC: breast cancer
CEACAM-1: carcinoembryonic antigen cell adhesion molecule 1
CRC: colorectal cancer
CTLA-4: cytotoxic T-lymphocyte-associated protein 4
DC: dendritic cell
DNMT: DNA methyltransferase
HAT: histone aceyltransferase
HDAC: histone deacetylase
HMGB1: high-mobility group protein B1
IC: Immune checkpoints
ICI: immune checkpoint inhibitors
LAG-3: Lymphocyte-activation gene 3
MDSC: myeloid-derived suppressor cells
miRNA: micro RNA
NK: natural killer cell
PBMC: peripheral blood mononuclear cells
PC: prostate cancer
PD-1: programmed cell death-1
PD-L1/2: programmed cell death-ligand 1/2
PS: phosphatidylserine
Teff: T effector cell
TET: ten-eleven translocation
TIGIT: T cell immunoreceptor with Ig and ITIM domains
TIM-3: T-cell T cell immunoglobulin and mucin-domain containing-3
TILs: tumor-infiltrating cells
TME: tumor microenvironment
Treg: T regulatory cell.
